# Impact of type-1 collagen hydrogel density on integrin-linked morphogenic response of SH-SY5Y neuronal cells†

**DOI:** 10.1039/d1ra05257h

**Published:** 2021-10-07

**Authors:** D. Merryweather, S. R. Moxon, A. J. Capel, N. M. Hooper, M. P. Lewis, P. Roach

**Affiliations:** Department of Chemistry, Loughborough University Leicestershire LE11 3TU UK p.roach@lboro.ac.uk; Division of Neuroscience and Experimental Psychology, School of Biological Sciences, Faculty of Biology, Medicine and Health, The University of Manchester, Manchester Academic Health Science Centre Manchester M13 9PL UK; National Centre for Sport and Exercise Medicine (NCSEM), School of Sport, Exercise and Health Sciences, Loughborough University Leicestershire LE11 3TU UK

## Abstract

Cellular metabolism and behaviour is closely linked to cytoskeletal tension and scaffold mechanics. In the developing nervous system functional connectivity is controlled by the interplay between chemical and mechanical cues that initiate programs of cell behaviour. Replication of functional connectivity in neuronal populations *in vitro* has proven a technical challenge due to the absence of many systems of biomechanical regulation that control directional outgrowth *in vivo*. Here, a 3D culture system is explored by dilution of a type I collagen hydrogel to produce variation in gel stiffness. Hydrogel scaffold remodelling was found to be linked to gel collagen concentration, with a greater degree of gel contraction occurring at lower concentrations. Gel mechanics were found to evolve over the culture period according to collagen concentration. Less concentrated gels reduced in stiffness, whilst a biphasic pattern of increasing and then decreasing stiffness was observed at higher concentrations. Analysis of these cultures by PCR revealed a program of shifting integrin expression and highly variable activity in key morphogenic signal pathways, such as mitogen-associated protein kinase, indicating genetic impact of biomaterial interactions *via* mechano-regulation. Gel contraction at lower concentrations was also found to be accompanied by an increase in average collagen fibre diameter. Minor changes in biomaterial mechanics result in significant changes in programmed cell behaviour, resulting in adoption of markedly different cell morphologies and ability to remodel the scaffold. Advanced understanding of cell–biomaterial interactions, over short and long-term culture, is of critical importance in the development of novel tissue engineering strategies for the fabrication of biomimetic 3D neuro-tissue constructs. Simple methods of tailoring the initial mechanical environment presented to SH-SY5Y populations in 3D can lead to significantly different programs of network development over time.

## Abstract 2

Cellular metabolism and behaviour is closely linked to cytoskeletal tension and scaffold mechanics.

## Graphical abstract

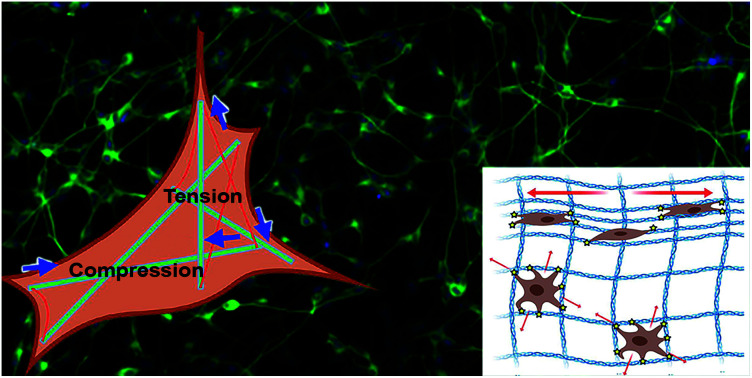

## 1. Introduction

The extracellular matrix (ECM) has been implicated as an active agent guiding cell behaviour and directing cell fate by local variations in scaffold composition and mechanics^1^,^2^. Matrix proteins are organized on the micro- and nano-scale to modify local mechanics and impart functional properties. Nervous tissues are among the softest found within the body and so present technical difficulties in handling and reproduction *in vitro*. Development of novel biomaterials that replicate this soft and highly organized matrix is lacking.^3^ Neuronal tissue engineering strategies currently seek to mimic this complex organization in 2D and 3D culture.^4^ In this paper collagen is explored as a model platform to explore the dynamic relationship between neuronal populations and the 3D organization of their environment during development *in vitro* as highlighted in previous work.^5^ Understanding the structural cues and programmed responses that guide cell migration, neuronal differentiation, and neurite outgrowth in 3D will provide further insights in the rational design of biomaterials for 3D neuronal tissue engineering.

In the central nervous system (CNS) mechanical properties remain poorly modelled, with observed range in Young's modulus of <1 to 20 kPa according to testing mode, inter-donor variability, and regional variations within the organ.^6^ Collagens are present throughout all layers of peripheral nerve ECM, organized to provide mechanical support to this delicate tissue with a tensile stress up to 10 MPa in human tibial and peroneal nerves.^7^ The ECM of the peripheral nerve has a complex 3D structure which is intrinsically implicated in nerve development and repair. Individual axons are protected within the endoneurium and grouped into fascicles by the perineurium, clustered to form a nerve fibre by the epineurium which encloses the whole nerve. At each level of organization, ECM components are arranged in space to generate mechanical support to resident glia and neuronal processes.^8^ Collagens of the epineurium are arranged into 10 to 20 μm thick oblique bundles that run parallel to the length of the nerve, straightening when tension is applied to provide protection from stretching injury.^9^ The perineural space is filled with a dense mesh of collagen fibres up to 3 μm in diameter, forming distinct cell-rich layers wrapping around neuronal fascicles. This connective layer is enriched with proteoglycans and elastic fibres forming a basal lamina for the axon that inhibits diffusion of proteins and ions.^10^ The endoneurium consists of two distinct layers of collagen, with a thin inner mesh layer directly enclosing each axon, and an outer layer of 1 to 3 μm thick collagen bundles parallel to the axis of the nerve. As in the epineurium, an oblique arrangement of endoneurium collagen fibres provides some protection from stretch injury, whilst application of tension to the nerve fibre induces an increase in collagen fibre alignment.^11^

Mechanosensitivity of cells is mediated by their adhesion to the ECM by integrin receptors. The role of mechanotransduction in biochemical signalling has been subject to intense study, implicating mechanical forces applied to the cytoskeleton in transcription regulation and nuclear arrangement^1^,^12^, regulation of cell differentiation^13^ and cell fate,^14^ as well as cell migration.^15^ Collagen concentration in 3D culture is a known regulator of neurite extension by variation of collagen inter-fibre spacing and hence mechanical stiffness, with higher concentrations resulting in greater numbers of collagen fibres present in a given volume, creating reduced inter-fibre spacing, increasing stiffness, and presenting greater impedance of growth cone pathfinding.^16^ Alternatively reduced inter-fibre spacing may create conflicting directional guidance cues that limit the strength of any one directional cue.

Integrins are a family of transmembrane cell adhesion proteins that transduce mechanical forces experienced by the cell matrix to an appropriate biochemical response by formation of the focal adhesion complex. Active integrins form an alpha–beta heterodimer complex, with 18 α-subunits and 8 β-subunits having been identified so far, producing 24 unique integrin heterodimers each specific to a number of molecular motifs.^17^ The majority of integrin-linked signalling machinery associates with the intracellular domain of the β-subunit,^18^ forming distinct strata with an integrin signalling focal adhesion kinase (FAK)-containing layer, a force transduction layer, and an actin regulatory layer that directly connects to the actin stress fibre.^19^ Upon binding to extracellular ligands, FAK is recruited to the intracellular domain of the β-integrin subunit in a mitogen-activated protein kinase (MAPK) dependent manner^20^ and the scaffold protein paxillin to FAK.^21^ Paxillin recruited by FAK then serves as the primary adaptor protein to which the majority of focal-adhesion associated signalling machinery is recruited.^22^ FAK–paxillin interaction is mediated by the degree of phosphorylation imparted by the MAPK/ERK signal pathway^23^ and by the mechanosensitive intermediary protein vinculin,^24^ with the mechanosensitive conformation of vinculin regulating the anchoring of MAPK1 and its proximity to FAK and paxillin within the focal adhesion complex.^25^

Physiological development and function of neuronal networks is closely linked to local mechanical environments and integrin-related signalling. Studies of neuronal stem cell fate commitment demonstrate a window as small as 24 hours in which matrix-stiffness regulation of transcriptional factors, β-catenin, and RhoA activity instruct cytoskeletal tension and differentiation program.^26,27^ Co-activation of integrin receptors alongside neurotrophin receptors is required to generate spontaneous firing in the developing brain and promote neuronal survival.^28^ Integrin activation and FAK activity regulate growth cone dynamics^29^ and neurite extension^30^ according to local mechanical properties,^31^ and has also been demonstrated as a significant factor regulating axonal growth within *in vitro* models.^32^

Integrin activity is intrinsically linked to many aspects of neuronal development *in vivo*. Understanding of integrin-related control of neuronal function *in vitro* is required to elucidate rational culture strategies to recapitulate neuronal development in 3D and understand how observed cell phenotypes relate to these molecular events stemming from integrin–ECM interactions. Collagen hydrogels are a well-studied platform from which neuronal response to mechanics have been explored,^16,33,34^ however the complex biochemical process of transduction to a phenotypic response has yet to be fully defined. Research into neuronal tissue form and function has historically been driven by animal-derived material, although humanisation of *in vitro* models has seen a large increase in human-derived cell lines such as SH-SY5Y. In this work we present a clear *in vitro* investigation of the impact of collagen hydrogel environment on a maturing SH-SY5Y neuronal network, and the changes imparted on the 3D biomaterial environment by the cells.

## 2. Materials and methods

All materials and chemicals were purchased from Thermo-Fisher Scientific and used as received, unless otherwise stated.

### 2.1 Cell culture protocol

Acid solubilized rat tail collagen type I at 2.05 mg mL^−1^ in 0.6% v/v acetic acid and 10× MEM (purchased from First Link, UK) was diluted with DMEM–GlutaMAX™ (Table 1) and neutralised with dropwise addition of 2 M sodium hydroxide. Upon neutralization pre-gel solutions were mixed with SH-SY5Y cells at a density of 1 × 10^5^ cells per mL and held at 4 °C for 5 minutes to allow initial fibrillogenesis to occur. 500 μL aliquots of cell-laden pre-gel solution were transferred to 24-well plates and incubated at 37 °C for 30 minutes to complete gelation of the collagen solution forming a disc 14 mm in diameter and 3 mm in depth. A needle was run around the outside of each well to detach the gel from the well wall. 500 μL growth medium comprising 10% foetal bovine serum (FBS), 1% penicillin–streptomycin (P/S) in DMEM–GlutaMAX™ was added to each well. Acellular gels were prepared in the same manner with the addition of acellular growth medium in place of a cell suspension. Control SH-SY5Y cultures were seeded in 24-well plates at a density of 5 × 10^4^ cells per well in 1 mL of growth medium. Cultures were maintained for 3 days in growth media. Half the media volume was replaced every day for 4 days with differentiation media consisting of 2% FBS, 1% P/S in DMEM–GlutaMAX™ with 2 μM all-*trans* retinoic acid, and 50 ng mL^−1^ brain-derived neurotrophic factor (BDNF).

**Table 1:** Components and volumes used to generate collagen hydrogel dilutions

Collagen dilution (mg mL^−1^)	Collagen solution/μL	MEM 10×/μL	DMEM/μL
1.78	850	50	100
1.00	476	50	474
0.50	238	50	712

### 2.2 Phase-contrast microscopy

Phase contrast images were collected on a Nikon TS2 microscope fitted with a Deltapix Invenio 3SII camera. For gel contraction analysis images of whole gels were captured within the 24-well plates. Culture plate images were captured in duplicate on days 1, 5, and 7 to track the diameter of each gel condition over the course of the culture period, with each gel being presented as triplicate repeats.

### 2.3 Immunohistochemistry

Cultures were fixed at day 3, prior to changing from growth to differentiation media, and day 7 (post differentiation) to assess relative expression and distribution of the neurofilament β-III-tubulin. Media was removed and samples were washed 3 times with PBS, before fixing samples with a 3.7% v/v formaldehyde solution in phosphate-buffered saline (PBS). Samples were washed and stored in PBS at 4 °C. Alternate washes with Tris-buffered saline (TBS) and deionized water (dH_2_O) were applied immediately before imaging. A blocking solution of TBS containing 5% v/v goat serum and 0.2% Triton X-100™ was applied for one hour. Primary staining of 0.5% v/v mouse anti-human-β-III tubulin and 0.5% goat serum in TBS was applied overnight. Samples were washed in alternate TBS and dH_2_O steps again, before secondary staining solution of 0.5% v/v Alexa Fluor™ 488-linked goat-derived anti-mouse antibody and 0.1% 4′,6-diamidino-2-phenylindole (DAPI) was applied for 3 hours at room temperature. Alternate washing steps were applied a final time immediately before imaging on a Nikon T2 inverted microscope fitted with a DynaCool camera. Filters used were ex/em 358/461 nm and 490/525 nm for DAPI and Alexa488 neurofilament imaging respectively. Three wells were imaged for each culture condition, with three images taken per well.

### 2.4 Cell viability assay

Cell viability was assessed at day 7 using a fluorometric cell viability assay kit (Abcam, UK) following manufacturer's protocols. Labelled wells were imaged on a Nikon T2 inverted microscope fitted with a DynaCool camera using ex/em 495/515 nm for the calcein-AM live stain and 528/617 for the ethidium homodimer. Three wells were imaged for each culture condition, with three images taken per well. Green and red fluorescence channels were overlaid to generate images for counting. All cells in each image frame were counted, with red-labelled cells counted as dead and green particles counted as live. Each sample was imaged to provide at least 3 regions of interest to provide a good overall assessment.

### 2.5 Gene expression analysis

Culture RNA was isolated from cell-laden hydrogels using an RNeasy RNA isolation kit (Qiagen). Hydrogels were first dissociated by repeated pipetting and RNA isolated according to the kit protocol. Complementary DNA (cDNA) was synthesized from extracted RNA by use of an iScript cDNA synthesis kit. Synthesized cDNA was included in a PCR reaction mix with OneStepPLUS SYBR Green Dye and custom PCR primers. Glyceraldehyde 3-phosphate dehydrogenase (GAPDH) was used as a housekeeping reference gene. GAPDH, ITGA1, ITGA2 and ITGB1 custom primers were purchased from Primer Design, and MAP3K3, FAK, ILK, and MMP-2 custom primers (Sigma-Aldrich). Relative expression of each gene to GAPDH was calculated by the double-delta *C*_*t*_ quantification method^35^ with cells cultured on tissue culture plastic used as a reference control group.

### 2.6 Measurement of gel mechanical remodelling

All gels were fixed in 3.7% v/v formaldehyde solution in PBS prior to analysis. After fixation gels mechanics were assessed in triplicate using a Discovery HR-2 rheometer (TA Instruments, UK) with a flat plate geometry, using a strain sweep, from 1 to 100% at a frequency of 10 rad s^−1^, to determine the linear viscoelastic region of the gel and assess its behaviour under stress-induced deformation of the molecular architecture of the gel, observed as a decline in the *G*′ modulus. Gels were subject to a frequency sweep from 1–10 rad s^−1^ to categorize stiffness. Reported moduli are taken as a mean average from the resistance of the gel at a plate rotational speed of 10 rad s^−1^.

### 2.7 Digital image analysis

All images were analysed using Fiji ImageJ processing package.^36^ Gel shrinkage was measured using the standard measure function, with values then normalized against the total diameter of the culture well. Cell morphology was assessed using the analyse particle function to quantify circularity, roundness, and aspect ratio of each cell with consideration that these measures are an approximation of 3D morphology from a 2D image.^37^ NeuronJ was used to measure total neurite length. Neurites were identified as thin projections extending from the cell body, brightly stained positive for β-III-tubulin. Length measurements were taken from the adjacent periphery of the cell nucleus, tracing the convoluted path of the neurite, either to its termination or until connecting to another cell body. Neurites longer than the highest confidence interval (∼120 μm) were classified as “long neurites” and their proportion within each experimental group calculated.

### 2.8 Statistical analysis

Graphing of collected data was carried out using Origin v2019. Data normality was assessed using D'Agostino *K*^2^ test. Statistical analysis was conducted by Student's *t*-test or ANOVA with bootstrapped Tukey's range test for normally distributed data with values of *p* < 0.05 considered significant. Error reporting in all plotted data represent ±1 standard deviation from the mean. Each assay was conducted in triplicate unless specified. Image analysis was conducted on three images taken of each experimental repeat, giving a total of nine images per group.

## 3. Results

### 3.1 Evolution of collagen hydrogel mechanics during SH-SY5Y culture

**Fig. 1:**
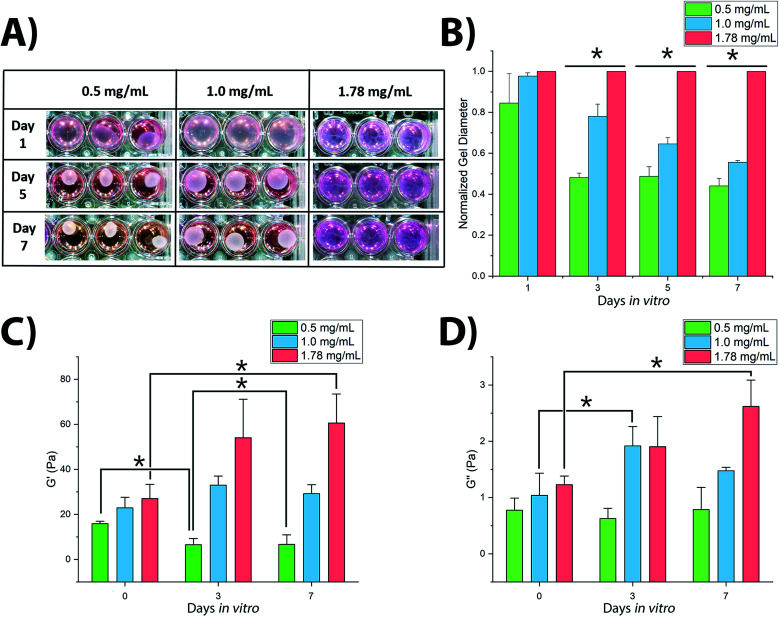
Variation in shape and mechanical characteristics of SH-SY5Y-seeded collagen hydrogels over culture period: (A and B) contraction of collagen gels and their measured diameters, *N* = 9; (C and D) rheological properties of the gels, *N* = 3. * = significant variance *p* > 0.05. Errors reported as ±st dev.

3D culture of SH-SY5Y cells within collagen hydrogels resulted in significant variations in hydrogel shrinkage over the course of the culture period as demonstrated by 2-way ANOVA of culture time and collagen concentration (*F*_(5,48)_ = 66.218, *p* < 0.001) (Fig. 1). Acellular gels were not observed to change in diameter over the course of the culture period (Fig. S1†). Significant differences in gel diameter were not observed by day 1 in culture (*F*_(2,15)_ = 3.004, *p* = 0.125), however gels were observed to shrink over later time periods, showing significant differences in terms of gel diameter: at day 3 (*F*_(2,15)_ = 300.921, *p* < 0.001), day 5 (*F*_(2,15)_ = 379.858, *p* < 0.001), and day 7 (*F*_(2,15)_ = 523.097, *p* < 0.001).

Rheological analysis of fixed gel samples indicated a complex relationship between gel contraction and gel stiffness, Fig. 1C and D. Acellular gels were found to increase in *G*′ storage modulus with increasing collagen concentration from 15.9 ± 1.0 Pa (0.5 mg mL^−1^) to 22.9 ± 4.7 Pa (1.0 mg mL^−1^), and finally to 27.0 ± 6.3 Pa (1.78 mg mL^−1^). This trend remained once inoculated with SH-SY5Y cultures, however cell culture in each gel condition resulted in significantly different changes in stiffness over time (*F*_(2,8)_ = 9.482, *p* = 0.01). In 0.5 mg mL^−1^ collagen gels, *G*′ reduced with culture time, Fig. 1C. The 1.0 mg mL^−1^ gels showed a biphasic evolution with *G*′ first increasing, before falling slightly by day 7. In contrast, *G*′ in 1.78 mg mL^−1^ gels was observed to increase significantly throughout the culture period. A similar relationship was observed in the *G*′′ loss modulus, Fig. 1D with 0.5 mg mL^−1^ collagen gels showing no significant change from day 0 to day 7, 1.0 mg mL^−1^ gels first increasing and then falling, and 1.78 mg mL^−1^ gels rising consistently across the 7 days. Calculation of the dynamic viscosity, assessing resistance to flow^38^ for each gel across the range of stress frequency, demonstrated a clear negative linear trend between 1 to 10 rad s^−1^ following the established pattern of evolution over the culture period according to the polymer concentration, as would be expected. These measurements are in good accordance with previously observed changes in collagen gel stiffness following culture of dorsal root ganglia^16^ and PC12 cells^39^ where increasing gel stiffness is associated with decreased neuronal migration, decreased neurite extension, and reduction in observed gel shrinkage.

### 3.2 Variance in SH-SY5Y morphology in collagen hydrogels

**Fig. 2:**
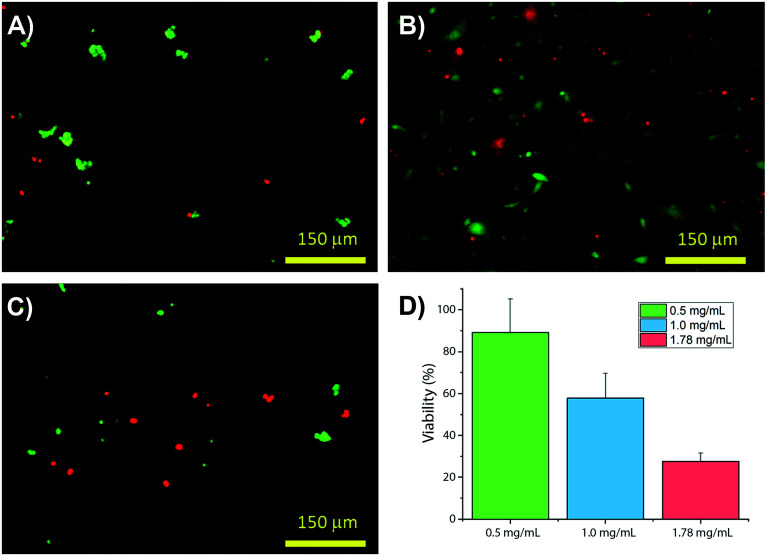
Changes in viability of SH-SY5Y populations in hydrogels with increasing collagen concentration: (A–C) present 0.5, 1.0 and 1.78 mg mL^−1^ collagen, respectively, plotted in (D) *N* = 3 for all samples measured with ±st dev.

Collagens are often used as a 3D matrix to support *in vitro* studies, with results here highlighting the impact of matrix density on the culture viability. Significant variation in cell viability at day 7 was observed between cultures (*F*_(2,8)_ = 12.703, *p* = 0.007), with viability decreasing with increased gel collagen concentration (Fig. 2). Viability was highest in 0.5 mg mL^−1^ collagen gels at 72.67 ± 16.07%. This was not significantly higher than in 1.0 mg mL^−1^ collagen gels displaying a mean viability of 44.27 ± 11.98% but was significantly higher (*p* = 0.005) than viability in 1.78 mg mL^−1^ gels, with a mean viability of 24.33 ± 4.03%.

**Fig. 3:**
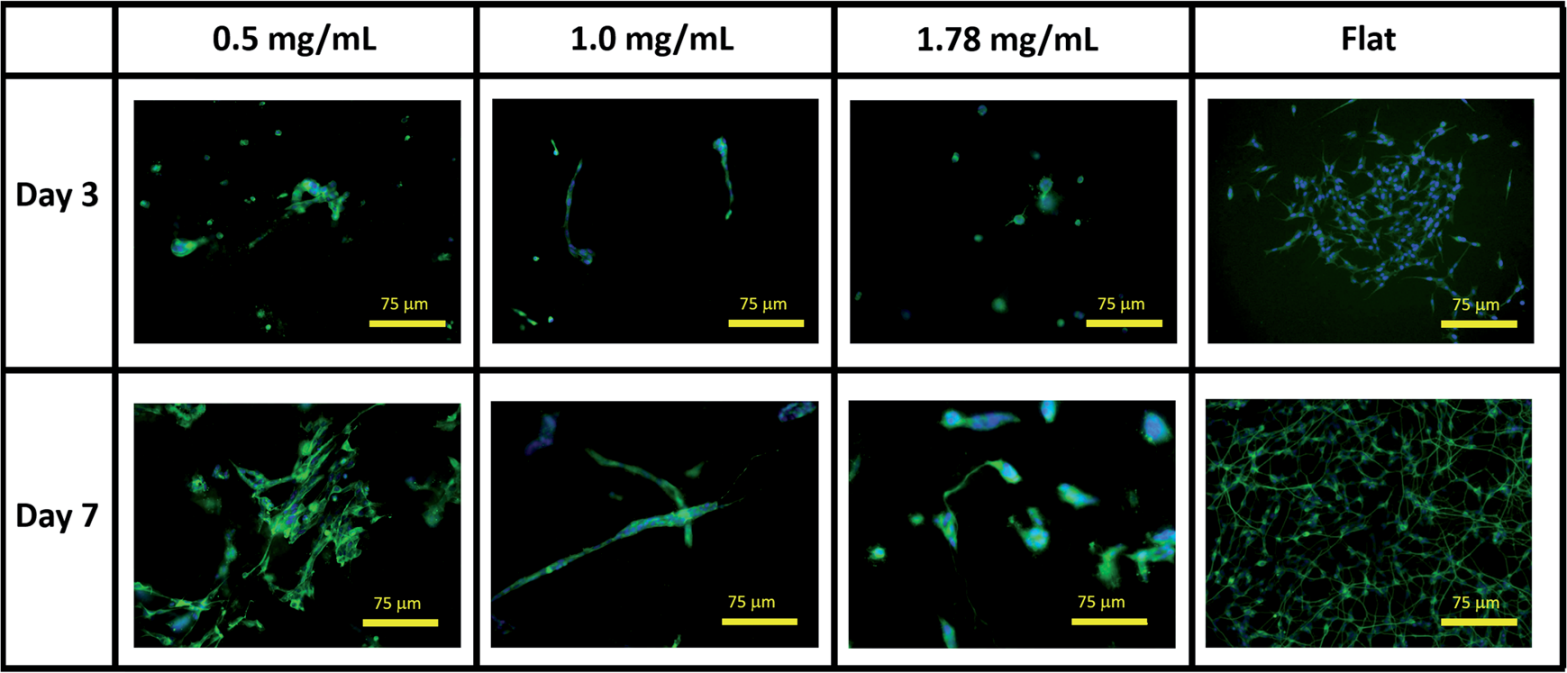
Representative immunofluorescent microscopy images of SH-SY5Y cultures in collagen hydrogel dilutions and on flat tissue culture plastic. Green = β_III_ tubulin, blue = DAPI.

**Fig. 4:**
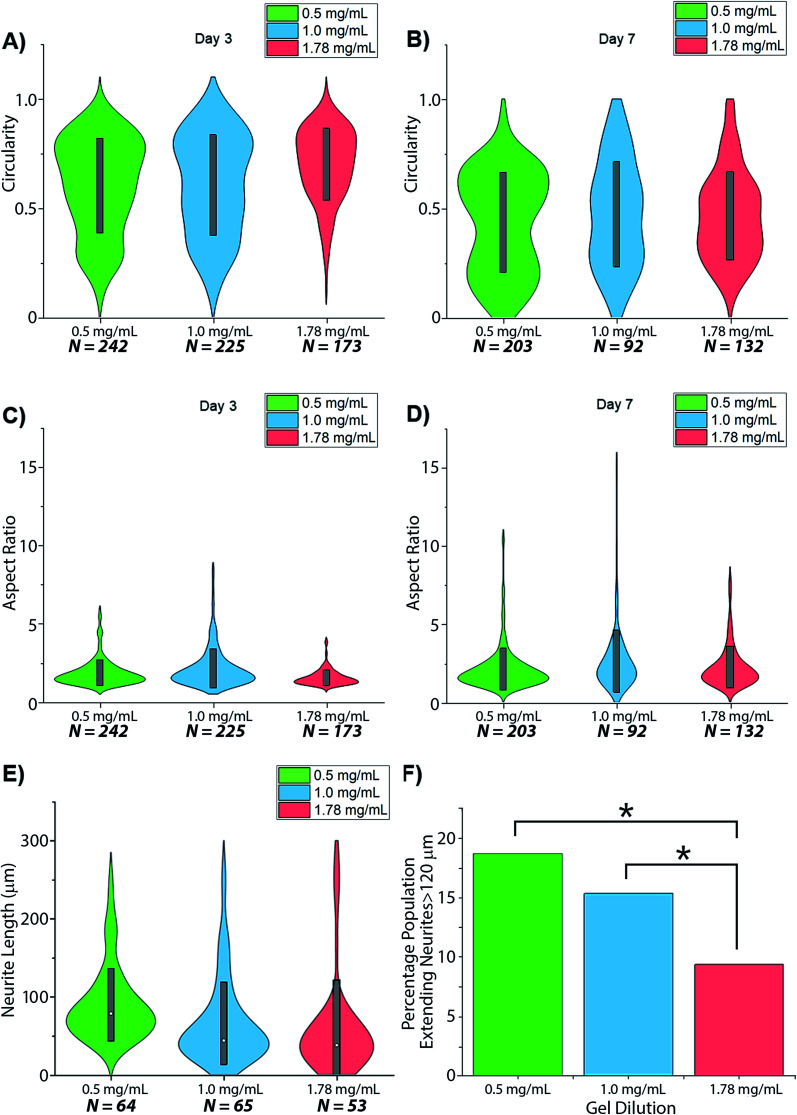
Changes in SH-SY5Y morphology in dilutions of collagen hydrogel. Morphological aspects reported as (A and B) circularity; (C and D) aspect ratio and (E and F) neurite length. * = significant variance *p* > 0.05.

Visual inspection of cell-seeded hydrogels over the course of the culture period readily showed a variety of cell morphologies adopted in response to local collagen concentration. In all gel conditions a wide range of cell morphologies were observed to form over time, with significant differences in the predominant morphology correlating to the collagen concentration of each gel condition. Cells displaying a range of morphologies were observed in all gels, Fig. 3, however the rate of change in cell circularity and cell aspect ratio was found to correlate with collagen gel density, Fig. 4. In each gel condition a population of neurons with an aspect ratio > 5 was observed to develop, with greatest frequency of these cells found within 1.0 mg mL^−1^ collagen gels. By day 3 such high aspect ratio cells were observed within 0.5 and 1.0 mg mL^−1^ gels, with the highest frequency occurring in 1.0 mg mL^−1^ conditions but were absent at 1.78 mg mL^−1^ collagen. By day 7 high aspect ratio cells were present in all gel conditions, with the highest frequency again present in 1.0 mg mL^−1^ conditions.

Neurites were not visibly extended in any gel condition by day 3. Neurite extension was found to be significantly greater by day 7 in 0.5 mg mL^−1^ gels relative to 1.0 mg mL^−1^ gels (*p* = 0.03) and 1.78 mg mL^−1^ gels (*p* = 0.005), with no significant difference identified in neurite length between 1.0 and 1.78 mg mL^−1^ gels (*p* = 0.747), Fig. 4E and F. In all conditions a morphology extending neurites above ∼120 μm (above 1 SD in the highest observed mean in 0.5 mg mL^−1^ gels) was observed with this morphology representing 18.75%, 15.38%, and 9.4% of the imaged cell population in 0.5, 1.0 and 1.78 mg mL^−1^ collagen gels respectively.

### 3.3 Analysis of SH-SY5Y metabolism in collagen gels

**Fig. 5:**
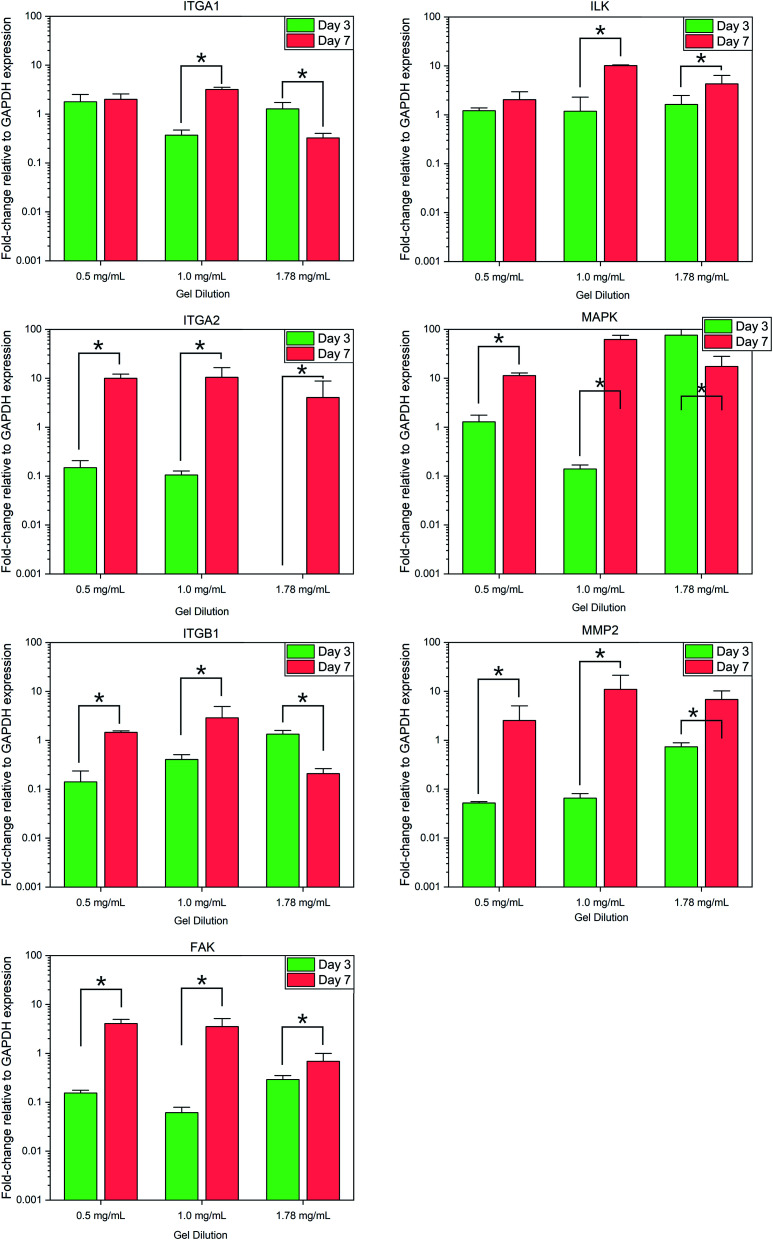
Fold-change in relative gene expression of target genes to the GAPDH housekeeping reference gene. * = significant variance *p* > 0.05, *N* = 3. Errors reported as ±st dev.

Normalized expression of the collagen specific integrins ITGA1, ITGA2, and ITGB1 demonstrated variances between each gel condition at each timepoint, with the change in expression between day 3 and day 7 also varying according to the collagen concentration of each gel, Fig. 5. Expression of ITGA1 rose from 1.78 ± 0.73-fold increase relative to flat controls to 2.00 ± 0.59 in 0.5 mg mL^−1^ collagen gels and from 0.37 ± 0.10 in 1.0 mg mL^−1^ gels. In contrast expression fell from 1.28 ± 0.44 to 0.32 ± 0.08 in 1.78 mg mL^−1^ gels. A similar trend was observed in the expression of ITGB1. Expression of ITGA2 was comparatively low in all culture conditions by day 3, falling below the detection threshold in 1.78 mg mL^−1^ collagen gels. Expression of this gene was observed to increase in all culture conditions by day 7.

Analysis of integrin-linked metabolic genes showed a less clear pattern in evolution. In general, expression increased from day 3 to day 7, however, the observed trend did not hold true for all genes analysed. Expression of FAK increased significantly in all time periods but the magnitude of this increase was lower in more concentrated gels, whereas an increase in ILK was much more significant in 1.0 mg mL^−1^ collagen gels.

Expression of MAP3K3 displayed variable trends in the evolution over the culture period according to culture conditions similar to that of integrin expression, with the most significant increase in 1.0 mg mL^−1^ collagen gels. Expression of the collagenase MMP2 was found to increase significantly (*F*_(1,17)_ = 8.479, *p* = 0.01) in all conditions from day 3 to 7.

## 4. Discussion

Neuronal differentiation, morphology, and neurite outgrowth are known to be closely linked to the machinery of cellular mechano-sensing,^39^ with control of neuronal cytoskeletal tension being a key regulator of axonal growth and development.^40^ Neuronal functionality is also closely linked to neuronal morphology, controlling the rate of propagation of action potentials across the cell soma.^41^ Here, we demonstrate a direct interplay between 3D cell scaffold characteristics and neuronal morphology, which causes downstream changes in cellular function. Assessment of integrin-related gene expression demonstrates a functional link between expression of integrins and integrin-linked kinases, the adoption of an elongated neuronal morphology, and gel scaffold shrinkage.

It is not uncommon to find that soft hydrogels will deform over culture time, with stresses imparted by attaching cells; often this is used within regenerative medicine strategies to align the collagen fibres into an axis parallel with the average forces.^42,43^ Here we have found that SH-SY5Y cells significantly reform collagen hydrogel networks, with higher gel concentrations of greater starting stiffness showing lesser change, Fig. 1. Cells were found to attach well in all cases, yet stiffer gels demonstrated lower viability over the culture period, Fig. 2. Cell morphology, being directly related to attachment and response to environmental cues, indicated significant differences across the gels over time. This highlights the dynamic feedback cycle of cell–surface interactions affecting the local cell environment, which in turn reciprocally impact on cell function and attachment *via* integrins.

**Fig. 6:**
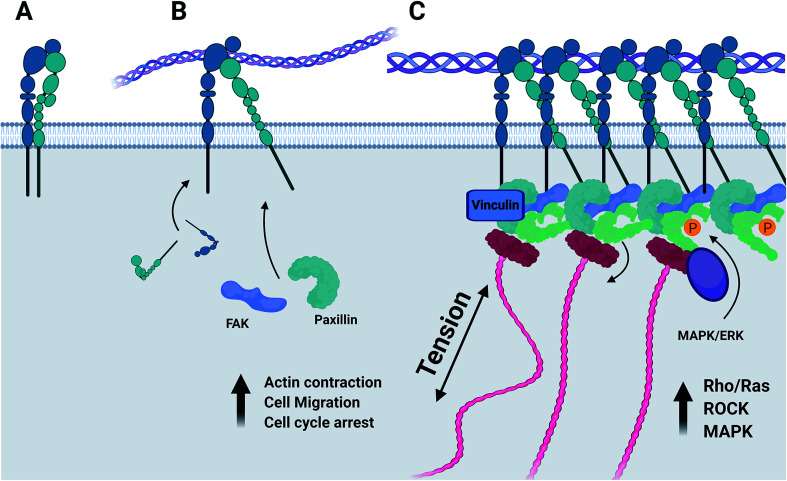
(A) Integrin dimers form at the cell membrane. (B) Activation of the integrin–ligand complex initiates the recruitment of focal adhesion kinase, paxillin, and further integrins to the nascent focal adhesion complex. (C) Integrins are associated with the actin cytoskeleton by linkers such as talin. The mechanosensitive vinculin serves as a binding site for ERK/MAPK which induces phosphorylation of FAK and paxillin, regulating the cellular response to integrin–ECM signalling. Produced in Biorender.

Whilst both the α1β1 and α2β1 integrin heterodimers specifically mediate cell adhesion to collagenous substrates, (Fig. 6) activation upon ligand binding of each induces variable responses to this chemical environment.^44^ It has been established that of the two heterodimers, α1β1 is far more promiscuous in its ligand specificity, binding to multiple collagen isoforms and other neuronal ECM constituents such as laminin, whilst the α2β1 dimer has a far higher specificity to type-I collagens and much lower binding affinity to laminin.^45^ The expression of α1 integrin is largely restricted to early stages of neurogenesis during development and early maturation of the CNS of model organisms.^46^ Expression of both integrin α1 and β1 is downregulated *in vitro* when maximal neurite outgrowth is achieved.^45^ Integrin α2 in contrast is distributed lightly through the mature CNS^47^ and is also localized to active nerve termini in the PNS.^48^ In terms of functionality, the α1β1 dimer is known to mediate functions of cell adhesion such as neurite outgrowth^49^ and plays a unique role regulating proliferation of cells during development *in vivo*.^50^ The α2β1 dimer has been implicated regulating the turnover of MMPs and focal adhesion complexes,^51^ in the formation of mature collagen fibrils by a RhoA-mediated cytoskeletal pathway,^52^ and in controlling contraction of free-floating collagen matrices.^53^

Variations in rate of gel shrinkage observed across the dilution range of collagen utilized here (Fig. 1) also mirror the expression of the two integrin dimers α1β1 and α2β1, Fig. 5. ITGA2 is upregulated significantly in all gel conditions by day 7, reflecting the reported role of the α2β1 dimer in forming mature isotropic collagen fibres, remodelled from a more diffuse polymer network of collagen fibrils.^54^ In contrast the α1β1 dimer is thought to primarily regulate cell migration and early-stages of remodelling.^55^ In lower gel dilutions integrin–ligand-binding results in contraction of the gel as collagen fibrils are manipulated by cytoskeletal forces and arranged in a suitable manner to support increasing cell polarity. As the gel polymer is reordered in this way, the number of interactions between anisotropic fibrils is reduced, resulting in decreased steric hindrance between these fibrils in response to rotational shearing forces, resulting in lower apparent *G*′ stiffness over time (Fig. 1). In contrast, at the higher collagen concentration of 1.78 mg mL^−1^ the initial polymer network is presumably sufficiently dense that adhered SH-SY5Y cells are unable to generate sufficient force along individual fibrils to manipulate the surrounding matrix. Instead, increased cell–matrix interactions are formed around the circular periphery of the cell (Fig. 3). The gel is contracted around the entirety of the cell membrane, forming small lacunae of increased density and resulting in increased apparent stiffness in response to rotational shear forces as the diffuse network is doped throughout by regions of increased stiffness around cells or cell clusters (Fig. 1C and D).

Cell morphology was shown to be responsive to hydrogel concentration, with circularity decreasing as the aspect ratio of cells increased, due to extension of neurites, Fig. 4. The comparative rate of neurite extension observed within fabricated type-I collagen hydrogel scaffolds reflects the biphasic response observed in the developing nerve *in vivo*^56^ and *in vitro* culturing explants of dorsal root ganglia within collagen gels,^16^ with maximal cellular elongation and neurite extension occurring at a midpoint in studied material stiffness. It is notable however that whilst neurite extension is mediated by integrin activation, this is required to sustain a chemotactic response.^57^ Decreased material stiffness within gel-like materials generally corresponds to greater porosity and increased inter-fibre spacing, restricting the physical space within and along which neurite extension might occur. Hence greater measured neurite outgrowth in lower concentration gels may result from restricted neurite pathfinding towards a local target that is more accessible within the dense polymer network presented to the cell at higher gel concentrations. Responses to changes in scaffold mechanics may then relate both to the implied structural forces imparted to the cytoskeleton as well as changes to the physical organization of the supporting scaffold in space, as observed across the multiple hierarchical layers of organization within biological structures such as the peripheral nerve fibre.

## 5. Summary and conclusions

The local environment presented to cells both *in vitro* and *in vivo* is of primary importance when considering scaffold technology, medical devices and tissue engineering strategies. Hydrogels present a desirable range of scaffold biomaterials used to support cells due to their ability to support 3D culture and distribute media nutrients throughout the volume. Interaction between cells and their scaffold impact hugely on internal cellular mechanics, largely *via* integrin-mediated control. This is particularly relevant for soft tissues such as the nervous system where small changes in biomaterial composition or presentation can have drastic effects on neural morphology, neurite extensions and overall tissue function. Here we have demonstrated the close connectivity between collagen gel concentration and development response of a model neuronal population, with higher collagen concentrations displaying lesser shrinkage during culture, lower cell viability, and directly linked integrin expression with key morphogenic signal pathways. Small variations in mechanical properties presented by the initial hydrogel result in profound variations in cell program downstream, demonstrating the genetic impact of biomaterial interactions *via* mechano-regulation. This work presents an accessible platform from which the influence of hydrogel mechanics and polymer organization have on the development of 3D neuronal networks can be explored *in vitro*, with relevance to the development of novel biomaterials and neuronal tissue engineering strategies.

## Conflicts of interest

There are no conflicts to declare.

## Supplementary Material

RA-011-D1RA05257H-s001
