# Supplementary material for: Impact of type-1 collagen hydrogel density on integrin-linked morphogenic response of SH-SY5Y neuronal cells
Source: RSC Adv. 2021 Oct 7;11(52):33124–35. doi: 10.1039/d1ra05257h (PMC9042137; doi:10.1039/d1ra05257h)
Supplement: RA-011-D1RA05257H-s001 [file RA-011-D1RA05257H-s001.pdf]

Electronic Supplementary Material (ESI) for RSC Advances.
This journal is © The Royal Society of Chemistry 2021

Supplementary Info

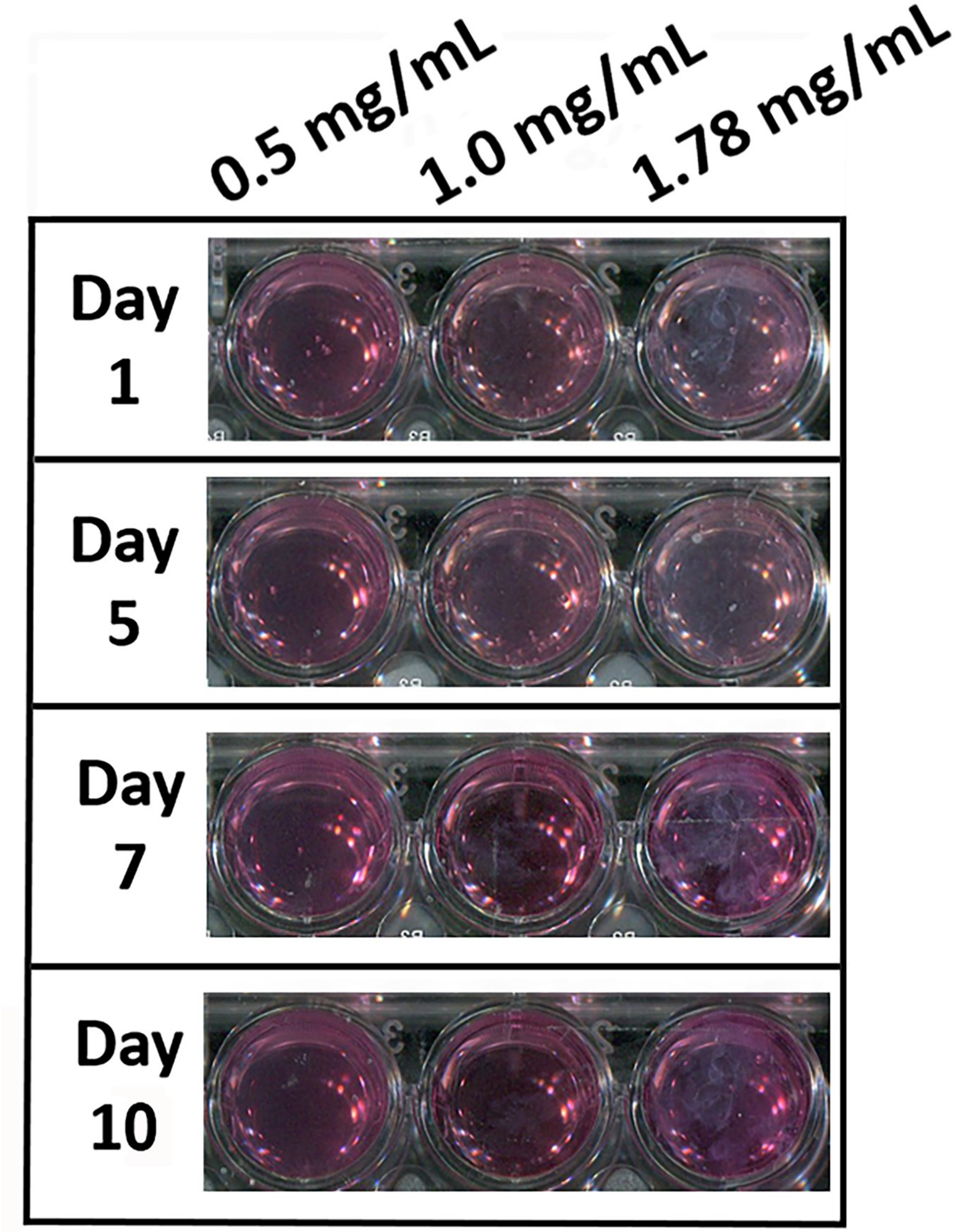

Figure S1: Highting no variation in shape of none cell-seeded collagen hydrogels over culture period. Extension of the time for gels to 10 days showed that no change in shape was observed.
